# Electron microscopy of human fascia lata: focus on telocytes

**DOI:** 10.1111/jcmm.12665

**Published:** 2015-08-27

**Authors:** Joanna Dawidowicz, Sylwia Szotek, Natalia Matysiak, Łukasz Mielańczyk, Krzysztof Maksymowicz

**Affiliations:** a Veterinary Clinic “Brynów”Katowice, Poland; b Faculty of Mechanical Engineering, Wrocław University of TechnologyWrocław, Poland; c School of Medicine with the Division of Dentistry in Zabrze, Department of Histology and Embryology, Medical University of SilesiaZabrze, Poland; d Faculty of Medicine, Wroclaw Medical UniversityWrocław, Poland

**Keywords:** human fascia lata, transmission electron microscopy, telocytes, mast cells, fibroblasts, myofibroblasts

## Abstract

From the histological point of view, fascia lata is a dense connective tissue. Although extracellular matrix is certainly the most predominant fascia’s feature, there are also several cell populations encountered within this structure. The aim of this study was to describe the existence and characteristics of fascia lata cell populations viewed through a transmission electron microscope. Special emphasis was placed on telocytes as a particular interstitial cell type, recently discovered in a wide variety of tissues and organs such as the heart, skeletal muscles, skin, gastrointestinal tract, uterus and urinary system. The conducted study confirmed the existence of a telocyte population in fascia lata samples. Those cells fulfil main morphological criteria of telocytes, namely, the presence of very long, thin cell processes (telopodes) extending from a relatively small cell body. Aside from telocytes, we have found fibroblasts, mast cells and cells with features of myofibroblastic differentiation. This is the first time it has been shown that telocytes exist in human fascia. Currently, the exact role of those cells within the fascia is unknown and definitely deserves further attention. One can speculate that fascia lata telocytes likewise telocytes in other organs may be involved in regeneration, homeostasis and intracellular signalling.

## Introduction

Fasciae are structures forming sheets beneath the skin, enveloping muscles and internal organs, supporting them and protecting from injury 1. The main role of the fascial system, however, is to reduce friction between muscles and transmit mechanical forces generated by the musculoskeletal system 2–4. Pathological processes affecting fasciae clinically manifest themselves as numerous different diseases, such as myofascial pain syndromes, Dupuytren’s contracture, congenital fascial dystrophy, compartment syndromes, hernias and fibromyalgia 5,6. Because the exact pathogenesis of the majority of these disorders remains unknown, they are often difficult in terms of diagnosis and treatment. On the other hand, as result of its durability, elasticity and relative ease of harvesting, the fascia, particularly the fascia lata, has a broad range of uses as a valuable graft material 7,8.

Recently, fascial structures have become an increasingly important subject of investigation among scientists as well as clinicians. There is a substantial body of research on fascia but it has generally focused on its gross anatomy, histology, the mechanical properties and chemical composition 9–17. However, the fascia ultrastructure with its characteristics of main cell populations has not been studied well enough 11,15. In particular, there is deficit of transmission electron microscopy investigations of cellular components of both superficial and deep fascial structures including the fascia lata.

This study was undertaken to broaden the knowledge about fascia lata cellular components using transmission electron microscopy techniques. Special emphasis was given on verification of telocytes presence. Telocytes are relatively recently discovered cells involved in a number of essential biological processes 18–20. Telocytes belongs to the group of stromal cells 21,22. They are present in the interstitial space of many human and animal tissues forming within the stromal compartment a unique, complex and integrative three-dimensional network 18,19,23. Telocytes are usually found in close vicinity to each other and to other cells, creating, homocellular or heterocellular junctions respectively (*e.g*. with endothelial cells, smooth muscle cells, stem cells, mast cells, eosinophils, adipocytes and fibroblasts) 20,24,25. Through this close contact, telocytes actively contribute to the maintenance of tissue homeostasis and play a role in tissue regeneration and repair 26–28. Moreover, they are involved in intracellular signalling by both formation of intracellular junctions as well as *via* a paracrine mode of action (secretion of soluble mediators such as interleukin-6, VEGF and nitric oxide) 21,28–31.

The telocytes are able to transport genetic material *via* the extracellular vesicles, and through this mechanism, they may regulate gene expression and the phenotype of the other cells (in particular stem cells) 32–34. So far, the presence of telocytes has been confirmed within different organs and tissues 20 but not yet in human fascial structures. Broadening our knowledge about distribution, ultrastructure and function of telocytes populating fascia lata may contribute to better understanding of the pathogenesis of fascial disorders and may help to optimize the use of fascia lata as a graft material.

## Materials and methods

The material consisted of samples of pathologically unchanged human fascia lata, which were collected post mortem from adult males (*n* = 7). This study was carried out in strict accordance with the Code of Ethics of the Declaration of Helsinki and with the recommendations of the Bioethics Committee of the Wroclaw Medical University (No. KB-262/2010).

Immediately after collection, the material was fixed in 2.5% glutaraldehyde in cacodylate buffer (pH 7.4) for 2 hrs at room temperature and then washed several times in the same buffer. After fixation, the samples were post-fixed in 1% OsO_4_ and dehydrated in increasing concentrations of ethanol and propylene oxide series. Subsequently, the samples were embedded in Epon 812 resin and polymerized for 48 hrs at 60°C. Ultrathin sections (70–100 nm) of white interference colour were cut on a Reichert OmU-3 ultramicrotome (Omu 3; Reichert, Vienna, Austria) equipped with a diamond knife (45°; RMC, Tucson, AZ, USA). The sections were mounted on 300 mesh copper grids and stained with 0.5% aqueous uranyl acetate and lead citrate using Leica EM AC 20 stainer (Leica Microsystems, Vienna, Austria). Subsequently, the grids were air-dried and examined under a TECNAI^–^ G2 12 Spirit BioTWIN transmission electron microscope (TEM; FEI, Eindhoven, the Netherlands) at 120 kV. Images from a number of randomly selected regions per sample were captured with a Morada CCD camera (Olympus Soft Imaging System Solutions GMBH, Münster, Germany). Morphometric analysis was performed with TEM Imaging and Analysis software (FEI). The number of cells were expressed as cell per field of view - FOV (7,065 mm^2^ - the surface of the TEM gird of 3 mm diameter). Some cellular components were digitally coloured on TEM images using Adobe© Photoshop CS3 to better evaluate their details.

## Results

Electron microscopic evaluation confirmed that fascia lata is a poorly cellular structure. Among fascial cellular components we found mainly fibroblasts, mast cells and cells showing features of myofibroblastic differentiation. Apart from these cells, cells with morphology similar to that of telocytes were observed.

**Figure 1:**
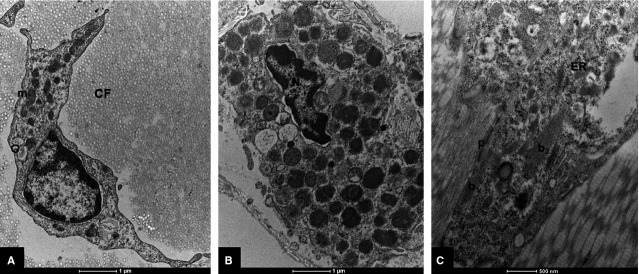
Electron micrograph of human fascia lata. (A) Fibroblast at lower magnification; not spindle-shaped cell situated within densely packed collagen fibres (transsections), abundant mitochondria (m) and cisterns of the Golgi apparatus (G); scale bar = 1 μm. (B) Mast cell filled with numerous secretory granules of different electron densities, sizes and shapes; note nucleus (N) with peripherally condensed chromatin; scale bar = 1 μm. (C) Section of cell showing the features of myofibroblastic differentiation; note the presence of myofilaments bundles at the cell periphery (b_1_) and also near the cell centre (b_2_), abundant rough endoplasmic reticulum (ER) and focally surface attachment plaques (p); scale bar = 0.5 μm. CF: collagen fibres.

The most numerous cell populations were represented by fibroblasts (3–6 cells/FOV per field; Fig. 1A). Observed mast cells (0–4 cells/FOV) were filled with various numbers of secretory granules of different electron densities, sizes and shapes, exhibiting mainly mixed scroll-homogeneous and homogenous substructural patterns (Fig. 1B).

Cells showing myofibroblastic differentiation were much less numerous (0–2 cells/FOV) compared to fibroblastic cell population. Their main feature was the presence of the intracytoplasmic smooth-muscle-type myofilaments abundant rough endoplasmic reticulum and surface attachment plaques (Fig. 1C).

**Figure 2:**
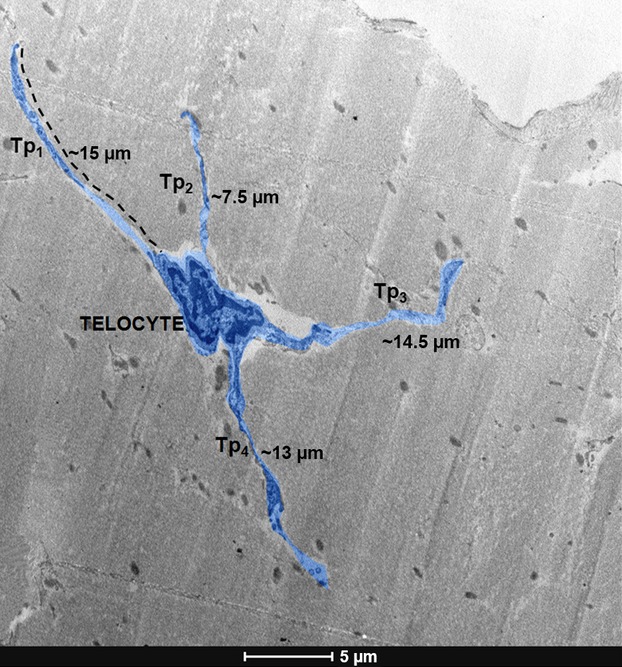
Digitally coloured transmission electron microscope (TEM) image (blue) of telocyte of human fascia lata; note relatively small cell body and 4 long characteristic processes - telopodes (Tp_1-4_); scale bar = 5 μm.

**Figure 3:**
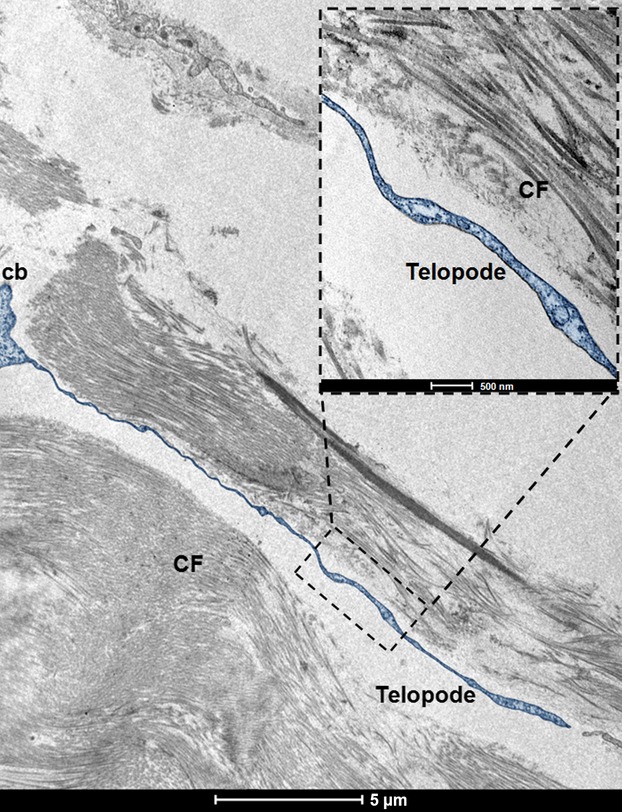
Digitally coloured (blue) electron micrograph of section of telocyte in human fascia lata; note the small part of the cell body (cb) and the very long telopode situated between collagen fibres (CF); scale bar = 5 μm. Red inset shows higher magnification of the part of telopode neighbouring collagen fibres (longitudinal sections); scale bar = 0.5 μm.

**Figure 4:**
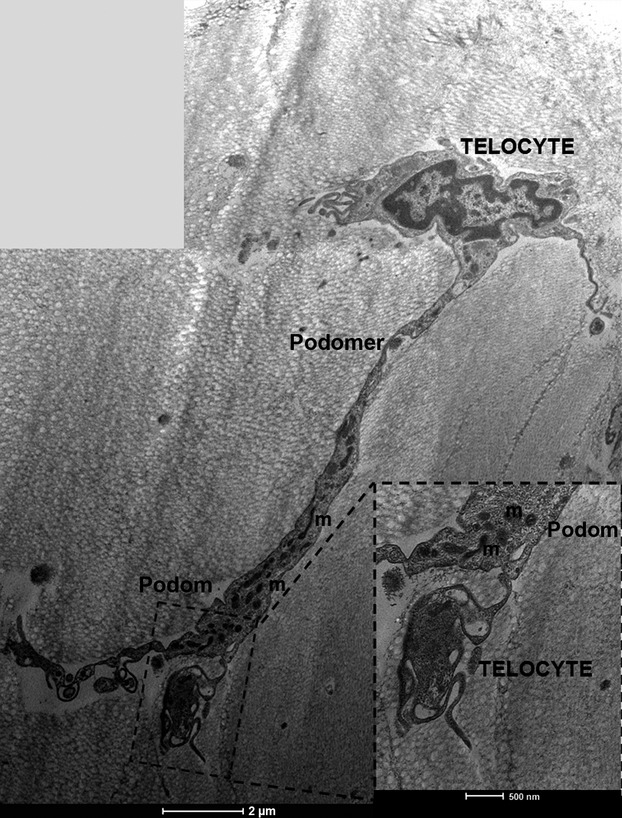
Transmission electron microscopy of telocytes within fascia lata; note large telopode extending from the cell body of telocyte with podomers alternating much thicker podoms containing abundant mitochondria (m); scale bar = 2 μm. Red inset shows a part of telocyte with telopode forming a circular, convoluted appearance; scale bar = 0.5 μm.

**Figure 5:**
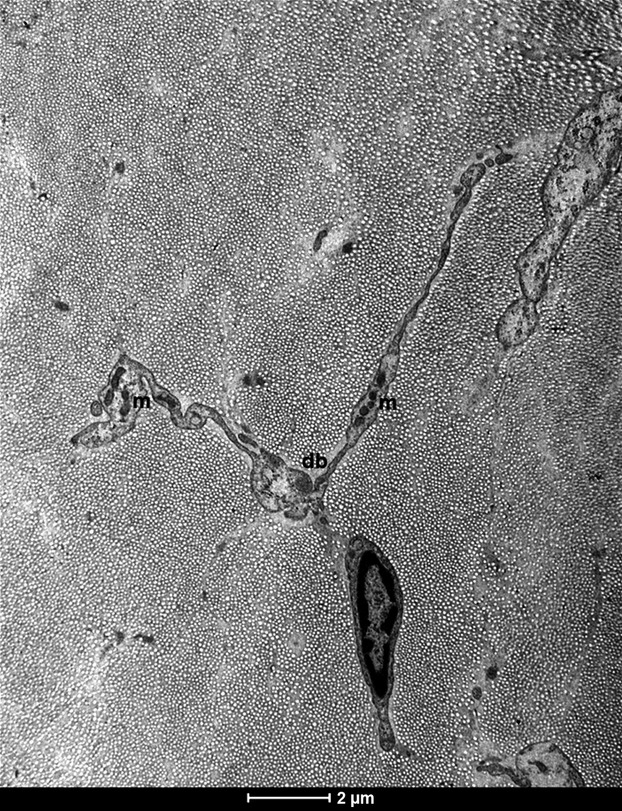
Electron micrograph of another telocyte in human fascia lata; note the telopode forming dichotomous branching (db) and focal accumulations of mitochondria (m) within podoms; scale bar = 2 μm.

**Figure 6:**
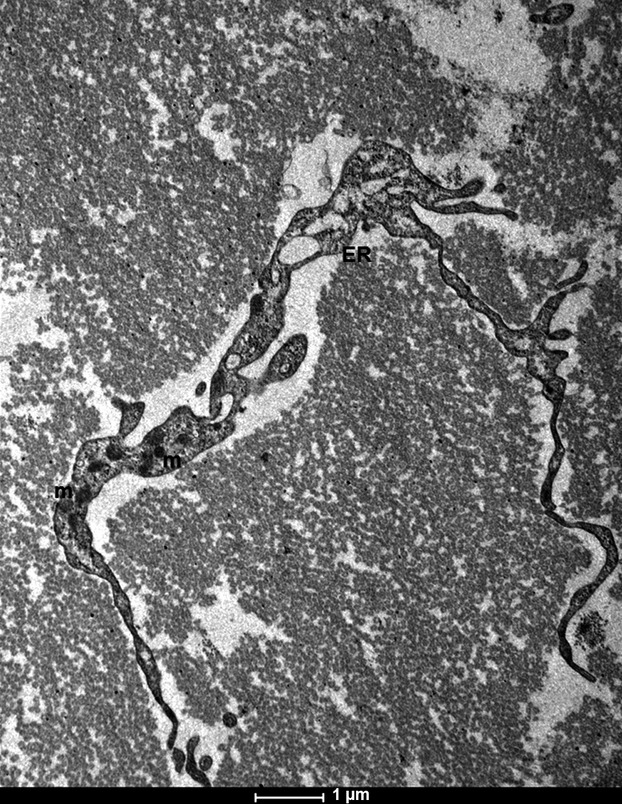
Electron micrograph of human fascia lata telocyte. Section of telopode with semi-circular arrangement and focal accumulations of mitochondria (m) and endoplasmic reticulum elements (ER); scale bar = 1 μm.

The observed telocytes (0–2 cells/FOV) had ultrastructural attributes of this distinct cell population. They exhibited the presence of typical, very long prolongations (telopodes). Each cell had several (up to 4) telopodes (Fig. 2), most often interdigitated among collagen fibres. The longest observed telopode measured about 22 μm in the place of the section (Fig. 3). The majority of telopodes was of variable thickness, with very thin segments (podomers) alternating with much thicker regions (podoms; Figs 4 and 5). The diameter of the observed podomers was slightly thicker compared to the thickness of collagen fibres (Fig. 3). Within some of the podoms, significant accumulations of mitochondria were present (Figs 6). In some areas, telopodes were forming a kind of semi-circular arrangement (Fig. 6) or dichotomous branching (Fig. 5). Sometimes fragments of telopodes were strongly convoluted (Fig. 4).

## Discussion

In the past, fasciae received little attention as their essential role in the proper functioning of the musculoskeletal system was not fully known and, consequently, underestimated. Today, fascia is no longer considered ‘a forgotten structure’. Recently, there has been a growing interest in fascial structures, with the number of publications rising each year 35. From the histological point of view, fascia lata, being a deep fascia of the lover extremity, represents (similarly to ligaments and tendons) mainly dense connective tissue 36. More precisely, it is composed primarily of collagen fibres, with much less numerous elastin fibres suspended within the ground substance. Although the extracellular matrix is certainly the most predominant component of the fasciae, there are also cell populations to be found within those structures 11,15. Fibroblasts play a crucial role in the formation and maintenance of fascial structure not only because these cells produce fibrous proteins (collagens, elastin) and ground substance components, but also because they are essential for modulating fascial architecture and controlling its stiffness 37. Mast cells are best known for their role in mediating allergic diseases. Moreover, they are involved in a number of other important processes, such as fibrogenesis and healing, which might be of great importance also with regard to fasciae 38. Myofibroblasts play a crucial role in establishing tension during wound healing and pathological contracture 39. However, it should be highlighted that even under physiological conditions, at rest, connective tissues seem to be under some sort of mechanical tension 39,40. It could be speculated that myofibroblasts are involved in the cellular basis of such tensional homeostasis thanks to the presence of a contractile apparatus that contains bundles of actin microfilaments 41–43. It is assumed that, because of the myofibroblast content, fasciae not only passively transduce mechanical forces, but are also able to contract in an active way 44,45.

Recently, telocytes have attracted a lot of attention from researchers 20. Those cells have been found in a number of different organs and tissues, such as in cardiac muscle, skeletal muscle, skin, the stomach, intestine, the gallbladder, bone marrow, the uterus and the urinary system but not yet in the fascia 20,46–52.

Telocytes are situated mainly in close vicinity to other stromal cells, blood vessels and nerve endings 20,53–55. Besides being involved in the intercellular signalling, tissue regeneration and reparation 20,26,49, telocytes may play a role in angiogenesis because they express antigenic marker CD34 and secrete VEGF 56,57. It was described that the number of telocytes is reduced in fibrotic areas of the heart, intestinal wall, skin as well as in different organs of patients suffering of systemic sclerosis 58,59. Telocytes - enwrapped in collagen bundles - were also found in fibrotic oviduct 24. This suggested a potential role of those cells in the process of fibrotic remodelling. A hypothesis exists that telocytes might be involved in controlling fibroblasts activation and that, consequently, loss of telocytes (confirmed in fibrotic tissues) might promote the differentiation of fibroblasts into profibrotic myofibroblasts 59. The above-mentioned telocyte function may also be of special interest with regard to research into fibrotic fascial diseases such as Dupuytren’s contracture or plantar fibromatosis and should be a subject of future investigations. Nevertheless, it should be underlined that in pathologically unchanged fascia lata samples, the observed telocytes were not affected, despite being surrounded by abundant, and sometimes tightly packed, collagen bundles. It would be interesting to document the presence and behaviour of telocytes also in tendons and ligaments, as those structures represent, similar to fascia lata, collagen-rich dance connective tissue 60.

Even though telocyte presence within a given tissue could be confirmed using several techniques, such as immunofluorescence, immunostaining or proteomics and genetic methods transmission electron microscopy remains a valuable diagnostic tool in identifying this cell population 34,61–63.

Telocytes, as observed in TEM images, exhibit a small, oval-shaped cellular body of average length of 9.39 μm ± 3.26 μm (min 6.31 μm; max 16.42 μm) with the nucleus occupying about 25% of the cell’s volume. Cytoplasm is generally sparse in the perinuclear region of the cell, although this region is rich in mitochondria 18.

A telocyte’s cytoplasm also contains a small Golgi complex, elements of rough and smooth endoplasmic reticulum, and cytoskeletal elements. However, the most characteristic morphological feature of telocytes is the presence of a different number (up to 5) of very long (up to hundreds of μm) processes-telopodes. Excepting the axons of some type of neurons, telopodes are probably the longest cellular prolongations in the human body. Podoms are dilated segments of telopodes, which harbour mitochondria, endoplasmic reticulum, and caveolae and alternate with podomers (thin telopode segments) 18. Telopodes usually form a complex network, because of their branching and overlapping, forming three dimensional convolutions. All aforementioned morphological features of telocytes are clearly visible under a TEM, which allows them to be distinguished from other kinds of cells, especially fibroblasts and neurons 49. Recently, FIB-SEM (Focused Ion Beam Scanning Electron Microscopes) tomography has been considered to be a very promising method of 3D telocyte imaging, however, this technique is much less freely available compared to traditional electron microscopy 57. With regard to immunodiagnostics of telocytes, there is still a lack of a highly specific antigen which could be considered a unique telocyte marker. Presently, double-labelled immunostaining, using CD34 together with PDGFR alpha or beta, c-kit, and vimentin are the most widely available antibody choices for detection of telocytes detection 57,64.

In conclusion, this study documented the scarce cellularity of human fascia lata and confirmed that telocytes exist in this tissue. It was also shown that despite the availability of wide range of modern visualization techniques transmission electron microscopy remains a particularly useful tool for examining this cell population, allowing visualization of all their ultrastructural attributes.

Currently, the exact role of telocytes within fascia is unknown. In the authors’ opinion, morphology, distribution and behaviour of the fascia lata telocytes should be a subject of further studies. It will contribute to better understanding of the role of fascial system in health and diseases. Additionally confirmation of the presence of a mast cell population is tantamount to the presence of active substances of theirs secretory granules. Some of which may, in the future, become molecular targets for treatment of fascial disorders in both human and veterinary medicine.
